# Pericardite Constritiva Idiopática com Estrutura Restritiva em "Duplo Anel Vertical" em Paciente com Estenose da Via de Saída do Ventrículo Direito

**DOI:** 10.36660/abc.20230576

**Published:** 2024-06-03

**Authors:** Ying Jiang, Haisong Bu

**Affiliations:** 1 Central South University Xiangya Hospital Department of Cardiovascular Surgery Changsha China Department of Cardiovascular Surgery - Xiangya Hospital - Central South University, Changsha – China

**Keywords:** Idiopática, Pericardite, Estenose da Via de Saída do Ventrículo Direito, Cirurgia

## Introdução

A pericardite constritiva é definida como um processo inflamatório das camadas fibrosas e serosas do pericárdio que leva ao espessamento pericárdico e à compressão das câmaras cardíacas, resultando em uma redução significativa da função cardíaca.^1^ A pericardite tuberculosa continua a ser a principal causa de pericardite constritiva em todo o mundo, especialmente nas áreas rurais remotas dos países orientais, onde o nível de cuidados e serviços de saúde é relativamente atrasado. Outras causas incluem pós cardiotomia, doenças do tecido conjuntivo, irradiação pós-mediastinal, uremia e doenças idiopáticas.^2^ Apresentamos aqui um caso raro de pericardite constritiva idiopática com estenose grave da via de saída do ventrículo direito (VSVD). O tecido espessado formou uma estrutura restritiva em "duplo anel vertical", envolvendo o sulco atrioventricular e os ventrículos o que levou à compressão das câmaras cardíacas.

## Relato de Caso

Uma mulher de 35 anos de uma aldeia remota no sul da China, que apresentou falta de ar moderada induzida por exercício e desconforto torácico intermitente (sem quaisquer características de angina), foi encaminhada ao nosso departamento. Seis meses atrás, por causa da respiração aguda induzida pelo exercício, ela foi submetida a um ecocardiograma local e foi diagnosticada com espessamento pericárdico e derrame pericárdico (leve), que foi aliviado após tomar diuréticos e, em seguida, tomar empiricamente medicamentos antituberculose por não ter teste cutâneo de tuberculose na vila. O exame físico em nosso hospital revelou que a temperatura corporal era de 36,5°C e os sinais vitais estavam estáveis. A pressão venosa central estava aumentada (25mmHg), com sopro sistólico e diminuição do som cardíaco. Não houve outros achados clínicos notáveis, nem histórico familiar de cirurgia e doença infecciosa após histórico médico e exame físico. Não houve anormalidades óbvias no exame laboratorial, como rotina de sangue (contagem de glóbulos brancos: 8,0x10^9), ESR (12mm/h), conjunto completo de imunidade ao reumatismo (Negativo) e teste cutâneo de tuberculose (diâmetro de endurecimento<2mm).

**Figura 1:**
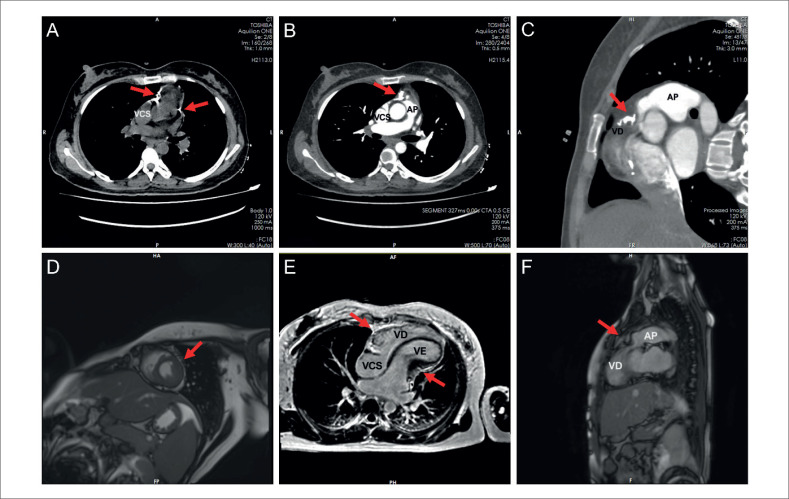
TC cardíaca demonstra espessamento e calcificação pericárdica e forma uma estrutura em anel transversal (A, setas); Compressão evidente da via de saída do ventrículo direito (B e C, setas); RM cardíaca mostrando pericárdio espessado (D, seta); A ressonância magnética confirmou a estrutura do anel transversal (E, setas) e a via de saída do ventrículo direito comprimida (F, seta). VCS: veia cava superior; AP: artéria pulmonar; VD: ventrículo direito; VE: ventrículo esquerdo.

Um ecocardiograma do nosso hospital revelou pericárdio espessado (especialmente para o sulco atrioventricular), veia cava inferior (VCI) alargada, estenose pulmonar e relaxamento ventricular limitado. Os diâmetros do VD, do ventrículo esquerdo (VE), da VSVD e da VCI foram 16 mm, 30 mm, 12 mm e 27 mm, respectivamente. A função sistólica do VE estava restrita com fração de ejeção de 55%. Regurgitações leves mitral e tricúspide também foram detectadas. Foi realizada tomografia computadorizada (TC) cardíaca que demonstrou espessamento e calcificação pericárdica ( Figura 1A e B , setas) e evidente compressão da VSVD ( Figura 1B e C , setas). O tecido espessado forma uma estrutura em anel transversal, que comprime a base ventricular esquerda e direita, resultando em deformação da cavidade cardíaca ( Figura 1A , setas). A ressonância magnética (RM) cardíaca também foi realizada para avaliar se ela afetava o miocárdio, e os resultados sugeriram que o pericárdio espessado, a estrutura do anel transversal ( Figura 1D e E , setas), comprimia a VSVD ( Figura 1F , seta).

**Figura 2:**
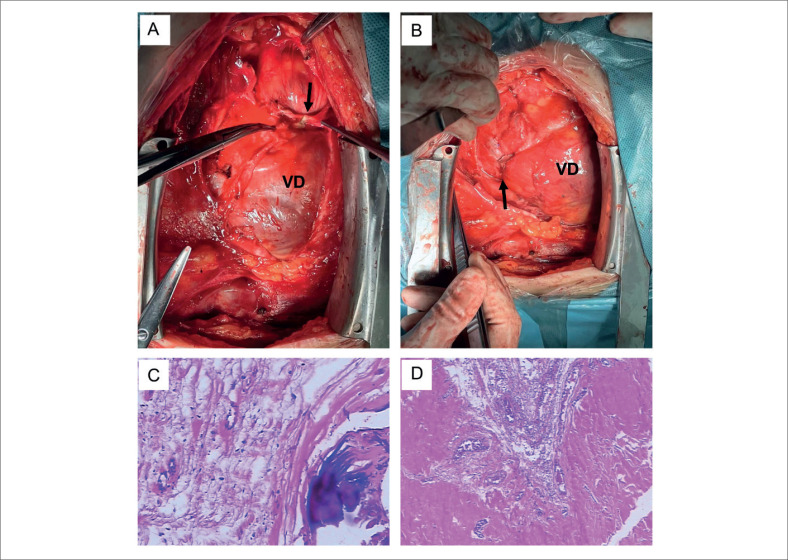
Visão intraoperatória da estrutura em "anel duplo vertical": a estrutura em anel transversal (A, seta); Corte da estrutura do anel mostrando a estrutura do anel sagital (B, seta); A imunohistoquímica no pós-operatório confirma o diagnóstico de pericardite constritiva com degeneração hialina e calcificação (C e D). VD: ventrículo direito.

**Figura 3:**
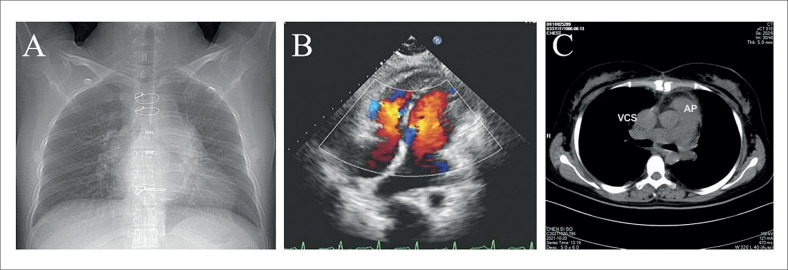
Radiografia de tórax pós-operatória (A), ultrassonografia cardíaca (B) e tomografia computadorizada (C) revelaram alívio da compressão da via de saída do ventrículo direito. VCS: veia cava superior; AP: artéria pulmonar.

Após extensas discussões com o paciente e sua família, foi agendada a remoção completa do pericárdio e a dissociação da estrutura restritiva em "duplo anel vertical" sem auxílio de circulação extracorpórea. Em caso de sangramento excessivo ou instabilidade hemodinâmica, o tratamento cirúrgico será realizado sob circulação extracorpórea. Primeiramente, o pericárdio foi incisado longitudinalmente com bisturi e, uma vez no plano correto, foi retirado primeiro do VD. O sangramento durante a dissecção pôde ser controlado por compressão suave com gaze úmida e quente ou suturas finas. Deve-se tomar cuidado extra para mobilizar e preservar os nervos frênicos. O achado intraoperatório foi que a estrutura do "anel duplo vertical" é composta pelo anel transversal na raiz da grande artéria e pelo anel sagital ao longo do sulco atrioventricular, limitando a contração cardíaca, especialmente na VSVD e na artéria pulmonar ( Figura 2A e B ). Após a ressecção do pericárdio atrial direito e ventricular direito, também é necessária a dissecção do pericárdio ao redor da aorta, artéria pulmonar, veia cava superior e VCI. A hemodinâmica permaneceu estável durante a operação, o limite da cavidade cardíaca estava claro e ativo após a operação, a estenose da VSVD foi significativamente aliviada ( Figura 2B ) e a pressão venosa central diminuiu para 13mmHg. Após cuidadosa hemostasia e fechamento da ferida por planos, o paciente foi cuidadosamente transferido para a unidade de terapia intensiva (UTI) em estado estável. Os espécimes removidos foram examinados patologicamente e não foram observados sinais de inflamação granulomatosa ou inflamação aguda, consistindo apenas de pericárdio espessado e fibrótico com calcificações ( Figura 2C e D ). O DNA bacteriano do complexo Mycobacterium tuberculosis e o gene *rpoB* relacionado à resistência à rifampicina não foram detectados na cultura histobacteriológica pós-operatória. O acompanhamento pós-operatório revelou alívio da compressão da VSVD ( Figura 3A - C ).

## Resultado e conclusão

O paciente recebeu alta hospitalar no 7° dia de pós-operatório, sem intercorrências, com retorno da pressão venosa central ao normal (12mmHg), com seguimento subsequente recomendado.

A principal causa da pericardite constritiva ainda é considerada a pericardite tuberculosa, que é definida como um processo inflamatório das camadas fibrosa e serosa do pericárdio.^1^ Fisiologicamente, o pericárdio aderente espessado diminui a complacência ventricular e restringe o enchimento cardíaco no final da diástole. O aspecto mais desafiador do diagnóstico da pericardite tuberculosa continua sendo estabelecer uma etiologia tuberculosa. Apesar dos nossos melhores esforços, cerca de 15% das doenças pericárdicas dificilmente são diagnosticadas,^3^ refletindo a escassez geral de novos testes de diagnóstico fiáveis e económicos que possam ajudar rapidamente na tomada de decisões clínicas. O resultado é que a prática em muitas regiões tem sido tratar a tuberculose empiricamente,^4^ especialmente nas áreas rurais remotas dos países orientais, onde o nível de cuidados e serviços de saúde é relativamente atrasado.

Os sintomas e sinais incluem fadiga, intolerância ao exercício, edema dos pés e, em casos extremos, síncope aos esforços, congestão hepática e ascite. Na ausculta, sons cardíacos abafados e/ou batida pericárdica são achados comuns. A radiografia de tórax pode mostrar calcificações pericárdicas e silhueta cardíaca normal em paciente com sintomas de insuficiência cardíaca direita. A ecocardiografia é a base de uma ferramenta diagnóstica não invasiva durante a triagem precoce^5^ que retrata o espessamento pericárdico, atividade ventricular, função cardíaca, etc. A TC e a RM fornecem excelentes informações adicionais para o diagnóstico. Além disso, oferecem avaliação tridimensional das relações anatômicas entre os grandes vasos e estruturas adjacentes^6^ e fornecem vistas seccionais das estruturas cardíacas de vários ângulos.

O manejo cirúrgico da pericardite constritiva envolve a remoção completa do pericárdio, que geralmente é realizada por meio de abordagem por esternotomia mediana. O tecido espessado e calcificado geralmente comprime os vasos sanguíneos e o coração. Como mostrado no presente caso, a estrutura em "anel duplo vertical" é extremamente rara, principalmente a estrutura em anel sagital, que se localiza no sentido do sulco atrioventricular. Felizmente, o paciente não apresentou estenose coronariana óbvia ou sintomas clínicos. Notavelmente, após a ressecção da estrutura em "duplo anel vertical", também é necessária a dissecção do pericárdio ao redor da aorta, artéria pulmonar, veia cava superior e VCI. É extremamente essencial observar que a dissociação excessiva do tecido fibroso e da lesão miocárdica no sulco atrioventricular deve ser evitada para evitar acidentes iatrogênicos. O corte parcial do tecido fibroso e o alívio da compressão devem ser realizados se uma extensa ressecção da estrutura do anel não puder ser realizada. Além disso, cortes histológicos adequados, patologia e cultura e detecção bacteriana são cruciais para um diagnóstico preciso.

No presente caso, o paciente apresentava suspeita de pericardite constritiva idiopática, incluindo estenose da VSVD e insuficiência cardíaca. Assim, foi realizada a remoção completa do pericárdio e dissociação da estrutura restritiva em "duplo anel vertical" para maximizar o benefício do paciente. Este relatório ilustrativo destaca a essência da melhoria do diagnóstico preciso, especialmente nas zonas rurais remotas dos países orientais, onde o nível de cuidados e serviços de saúde é relativamente atrasado. A falha em identificar a pericardite constritiva não tuberculosa neste caso atrasará o diagnóstico preciso e o tratamento eficaz. O acompanhamento com ecocardiografia e TC deve ser continuado para detectar recorrência e efeitos em longo prazo.
