# Supplementary material for: Pericardite Constritiva Idiopática com Estrutura Restritiva em "Duplo Anel Vertical" em Paciente com Estenose da Via de Saída do Ventrículo Direito
Source: Arq Bras Cardiol. 2024 Jun 3;121(5):e20230576. [Article in Portuguese] doi: 10.36660/abc.20230576 (PMC11164434; doi:10.36660/abc.20230576)
Supplement: Translation

# Translation: Idiopathic Constrictive Pericarditis with Restrictive "Vertical Double Ring" Structure in a Patient with Right Ventricular Outflow Tract Stenosis

**DOI:** 10.36660/abc.20230576i

**Authors:** Ying Jiang, Haisong Bu

**Affiliations:** 1 Changsha China Department of Cardiovascular Surgery - Xiangya Hospital - Central South University, Changsha – China

**Keywords:** Idiopathic, Pericarditis, Right Ventricular Outflow Tract Stenosis, Surgery

## Introduction

Constrictive pericarditis is defined as an inflammatory process of the fibrous and serous layers of the pericardium that leads to pericardial thickening and compression of the cardiac chambers, ultimately resulting in a significant reduction in cardiac function.^1^Tuberculous pericarditis remains the leading cause of constrictive pericarditis worldwide, especially in the remote rural areas of eastern countries where the level of health care and services is relatively backward. Other causes include postcardiotomy, connective tissue diseases, postmediastinal irradiation, uremia, and idiopathic.^2^Herein, we present a rare case of idiopathic constrictive pericarditis with severe right ventricular outflow tract (RVOT) stenosis. The thickened tissue formed a restrictive "vertical double ring" structure, involving the atrioventricular sulcus and ventricles that led to compression of the cardiac chambers.

## Case Report

A 35-year-old female from a remote village in southern China, who experienced moderate exercise-induced shortness of breath and intermittent chest discomfort (without any angina characteristics), was referred to our department. Six months ago, because of exercise-induced acute breathing, she underwent a local echocardiogram and was diagnosed with pericardial thickening and pericardial effusion (mild), which was relieved after taking diuretics, and then empirically taking anti-tuberculosis medications because there was not tuberculosis skin testing in the village. Physical examination in our hospital revealed that the body temperature was 36.5°C and the vital signs were stable. The central venous pressure was increased (25mmHg), with systolic murmur, and decreased heart sound. There were no other notable clinical findings and no family history of surgery and infectious disease following medical history and physical examination. There were no obvious abnormalities in laboratory examination, such as blood routine (white blood cell count: 8.0x10^9), ESR (12mm/h), complete set of rheumatism immunity (Negative), and tuberculosis skin test (hardening diameter<2mm).

An echocardiogram from our hospital revealed a thickened pericardium (especially for the atrioventricular sulcus), widened inferior vena cava (IVC), pulmonary stenosis, and limited ventricular relaxation. The diameters of the right ventricle (RV), the left ventricle (LV), the RVOT, and IVC were 16mm, 30mm, 12mm, and 27mm, respectively. The LV systolic function was restricted with an ejection fraction of 55%. Mild mitral and tricuspid regurgitation were also detected. Cardiac computed tomography (CT) was performed and demonstrated pericardial thickening and calcification ( Figure 1A and B , arrows), and obvious compression of the RVOT ( Figure 1B and C , arrows). The thickened tissue forms a transverse ring structure, which compresses the left and right ventricular base, resulting in cardiac cavity deformation ( Figure 1A , arrows). Cardiac magnetic resonance imaging (MRI) was also performed to assess whether it affected the myocardium, and the results suggested that the thickened pericardium, the transverse ring structure ( Figure 1D and E , arrows) compressed the RVOT ( Figure 1F , arrow).

**Figure 1:** Cardiac CT showing pericardial thickening and calcification and forms a transverse ring structure (A, arrows); Obvious compression of the right ventricular outflow tract (B and C, arrows); Cardiac MRI showing the thickened pericardium (D, arrow); MRI confirmed the transverse ring structure (E, arrows) and compressed right ventricular outflow tract (F, arrow). SVC: superior vena cava; PA: pulmonary artery; RV: right ventricle; LV: left ventricle.

After extensive discussions with the patient and his family, the complete removal of the pericardium and the dissociation of the restrictive "vertical double ring" structure was scheduled without the assistance of a cardiopulmonary bypass. In case of excessive bleeding or hemodynamic instability, surgical treatment would be carried out under cardiopulmonary bypass. First, the pericardium was incised longitudinally with a scalpel and once in the correct plane, it was removed first from the right ventricle. Bleeding during dissection could be controlled by gentle compression with warm wet gauze or fine sutures. Extra care must be taken to mobilize and preserve the phrenic nerves. Intraoperative finding was that the "vertical double ring" structure is composed of the transverse ring at the root of the great artery and the sagittal ring along the atrioventricular sulcus, limiting the cardiac contraction, especially at RVOT and pulmonary artery ( Figure 2A and B ). After the resection of the right atrial and right ventricular pericardium, the dissection of the pericardium around the aorta, pulmonary artery, superior vena cava, and IVC is also necessary. The hemodynamics were stable during the operation, the cardiac cavity boundary was clear and active after the operation, the RVOT stenosis was significantly relieved ( Figure 2B ), and the central venous pressure decreased to 13mmHg. After careful hemostasis and closing of the wound in layers, the patient was carefully transferred to the intensive care unit (ICU) in stable condition. The removed specimens were examined pathologically and were noted to have no evidence of granulomatous inflammation, or acute inflammation, consisting solely of the thickened and fibrotic pericardium with calcifications ( Figure 2C and D ). The bacterial DNA of the mycobacterium tuberculosis complex and rifampicin resistance-related *rpoB* gene were not detected in postoperative histobacteriological culture. Postoperative follow-up revealed relief of RVOT compression ( Figure 3A - C ).

**Figure 2:** Intraoperative view of the "vertical double ring" structure: the transverse ring structure (A, arrow); Cutting the ring structure section showing sagittal ring structure (B, arrow); Immunohistochemistry post-operatively confirms the diagnosis of constrictive pericarditis with hyaline degeneration and calcification (C and D). RV: right ventricle.

**Figure 3:** Postoperative chest X-ray(A), cardiac ultrasound(B), and CT(C) revealed relief of right ventricular outflow tract compression. SVC: superior vena cava; PA: pulmonary artery.

## Result and conclusion

The patient was discharged uneventfully on the 7th postoperative day with the central venous pressure returned to normal (12mmHg) with recommended subsequent follow-up.

The main cause of constrictive pericarditis is still considered to be tuberculous pericarditis, which is defined as an inflammatory process of the fibrous and serous layers of the pericardium.^1^Physiologically, the thickened adherent pericardium decreases ventricular compliance and restricts cardiac filling in late diastole. The most challenging aspect of the diagnosis of tuberculous pericarditis remains to be establishing a tuberculous etiology. Despite our best efforts, about 15% of pericardial diseases are hardly diagnosed,^3^reflecting the overall paucity of reliable, cost-effective new diagnostic tests that could rapidly aid clinical decision-making. The result is that the practice in many regions has been to treat tuberculous empirically,^4^especially for the remote rural areas of eastern countries where the level of health care and services is relatively backward.

Symptoms and signs include fatigue, exercise intolerance, pedal edema, and in extreme cases syncope on exertion, hepatic congestion, and ascites. On auscultation, muffled heart sounds, and/or a pericardial knock are common findings. The chest X-ray may show pericardial calcifications and a normal cardiac silhouette in a patient with right-sided heart failure symptoms. Echocardiography is the mainstay of a non-invasive diagnostic tool during the early screening^5^that depicts the pericardial thickening, ventricular activity, cardiac function, etc. CT and MRI provide excellent additional information for the diagnosis. Additionally, it offers a three-dimensional assessment of the anatomic relations between great vessels and adjacent structures,^6^and it provides sectional views of the cardiac structures from various angles.

The surgical management of constrictive pericarditis involves the complete removal of the pericardium, which is usually performed through a midline sternotomy approach. Thickened and calcified tissue usually compresses blood vessels and the heart. As shown in the present case, the "vertical double ring" structure is extremely rare, especially for the sagittal ring structure, which is along the direction of the atrioventricular sulcus. Fortunately, the patient had no obvious coronary stenosis or clinical symptoms. Notably, after the resection of the "vertical double ring" structure, the dissection of the pericardium around the aorta, pulmonary artery, superior vena cava, and IVC is also necessary. It is critically essential to note that excessive dissociation of fibrous tissue and myocardial injury at the atrioventricular sulcus should be avoided to avoid iatrogenic accidents. Cut off partial fibrous tissue and relieving compression should be performed if an extensive range of the ring structure resection cannot be performed. What is more, adequate histologic sections, pathology, and bacterial culture and detection are crucial for accurate diagnosis.

In the case herein, the patient had suspected idiopathic constrictive pericarditis, including RVOT stenosis and cardiac insufficiency. Thus, the complete removal of the pericardium and dissociation of the restrictive "vertical double ring" structure was performed to maximize the patient's benefit. This illustrative report highlights the essence of improving accurate diagnosis, especially for the remote rural areas of eastern countries where the level of health care and services is relatively backward. Failure to identify non-tuberculous constrictive pericarditis in this case will delay accurate diagnosis and effective treatment. Follow-up with echocardiography and CT should be continued to detect recurrence and long-term effects.
